# A Multi-Parameter Persistence Algorithm for the Automatic Energy Calibration of Scintillating Radiation Sensors

**DOI:** 10.3390/s25154579

**Published:** 2025-07-24

**Authors:** Guglielmo Ferranti, Chiara Rita Failla, Paolo Finocchiaro, Alessandro Pluchino, Andrea Rapisarda, Salvatore Tudisco, Gianfranco Vecchio

**Affiliations:** 1 Department of Physics and Astronomy, University of Catania, Via S. Sofia 64, 95123 Catania, Italy; guglielmo.ferranti@phd.unict.it (G.F.); failla@lns.infn.it (C.R.F.); alessandro.pluchino@ct.infn.it (A.P.); andrea.rapisarda@ct.infn.it (A.R.); 2 Istituto Nazionale di Fisica Nucleare, Sezione di Catania, Via S. Sofia 64, 95123 Catania, Italy; 3 Istituto Nazionale di Fisica Nucleare, Laboratori Nazionali del Sud, Via S. Sofia 62, 95123 Catania, Italy; tudisco@lns.infn.it (S.T.); vecchio@lns.infn.it (G.V.); 4 Complexity Science Hub, Metternichgasse 8, 1030 Wien, Austria

**Keywords:** peak detection, automatic energy calibration, topological data analysis, multi-parameter persistence, persistent homology

## Abstract

Peak detection is a fundamental task in spectral and time-series data analysis across diverse scientific and engineering disciplines, yet traditional approaches are highly sensitive to the choice of algorithm parameters, complicating reliable and consistent interpretation. Triggered by the requirement for the energy calibration for the 128 detectors of the PI3SO gamma ray scanner, we introduce a versatile methodology inspired by concepts from persistent homology, extending the traditional notion of persistence to a multi-parameter setting. Our approach systematically explores the space defined by multiple detection parameters and quantifies peak robustness through the hyper-volume in the parameter space where each peak is consistently identified. This volumetric multi-parameter persistence (VM-PP) measure enables robust peak ranking and significantly reduces the sensitivity of detection outcomes to individual parameter selection, demonstrating utility across simulated and experimental spectral datasets. Extensive validation reveals that this method reliably differentiates genuine peaks from noise-induced fluctuations under diverse noise conditions, proving effective in practical spectroscopic calibration scenarios. This framework, general by design, can be readily adapted to diverse signal-processing applications, enhancing interpretability and reliability in complex feature-detection tasks.

## 1. Introduction

Peak detection is a fundamental task in signal processing, underpinning applications in spectroscopy, mass spectrometry, astrophysics, and biomedical signal analysis [1,2]. This study was specifically motivated by the challenge of performing energy calibration for the 128 scintillation detectors of the PI3SO gamma-ray scanner [3]. The Proximity Imaging System for Sort and Segregate Operations (PI3SO) is an advanced gamma-ray spectroscopic imaging system developed to enhance the characterization and sorting of radioactive waste. It is specifically designed to identify radioactive hotspots within waste drums, facilitating the management of intermediate-level waste (ILW), low-level waste (LLW), and very low level waste (VLLW) [4,5]. The PI3SO system comprises two arrays of 64 thallium-doped cesium iodide (CsI(Tl)) scintillators, each measuring 1 × 1 × 1 cm^3^, optically coupled to 6 × 6 mm^2^ silicon photomultipliers (SiPMs) via optical grease with intermediate refractive index, enabling high-sensitivity gamma-ray detection. This configuration allows for high-spatial-resolution imaging and spectroscopic analysis of gamma-ray emissions. By automating the identification and localization of radioactive sources, PI3SO significantly reduces human intervention and exposure, while improving the accuracy and speed of the waste characterization process.

In this work, PI3SO’s detector array serves as the basis for developing and validating a novel peak detection and energy calibration methodology, aimed at automating the processing of gamma-ray spectra collected during radioactive waste sorting operations.

**Figure 1:**
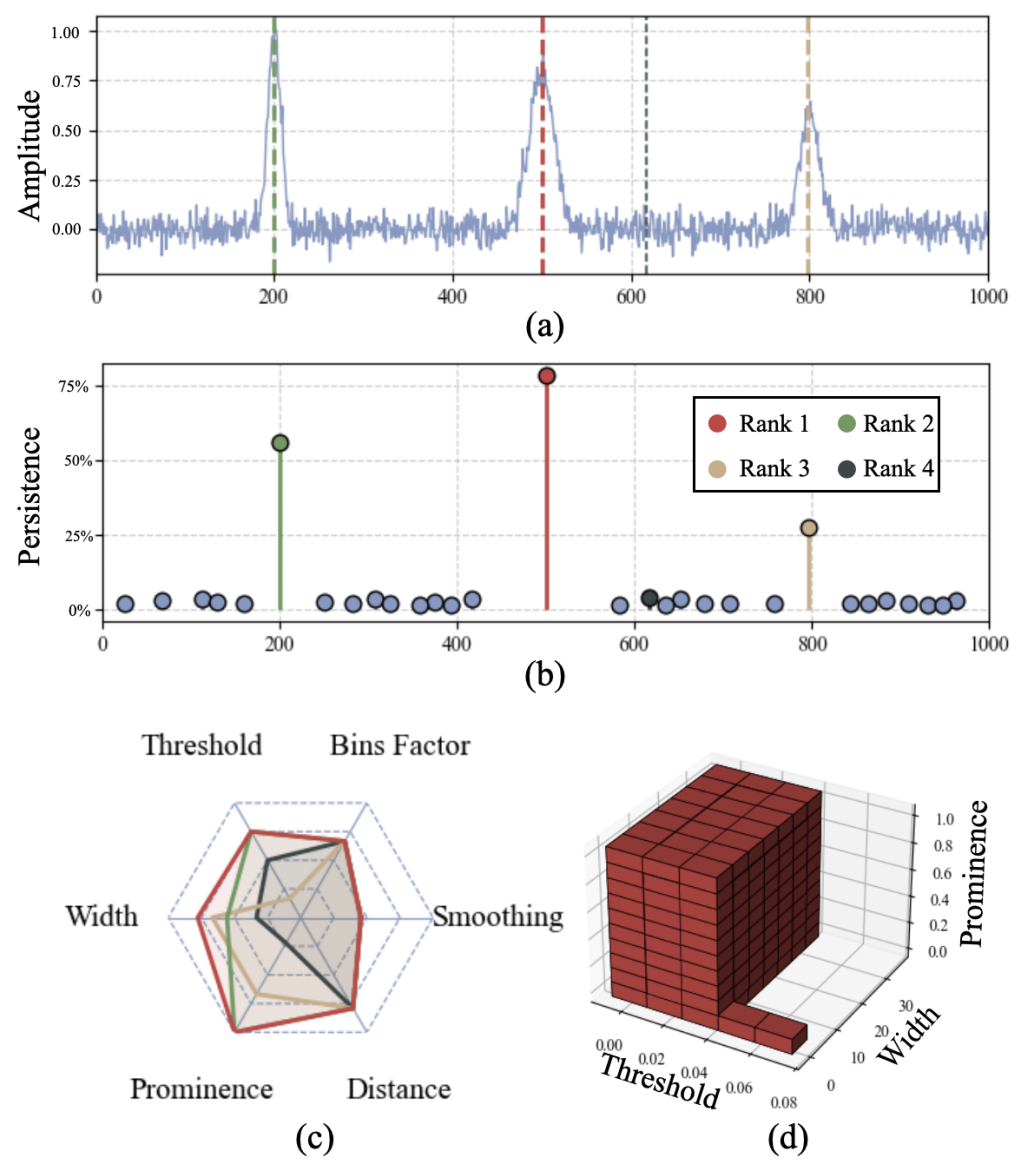
(**a**) Simulated signal, obtained by adding a bounded-range noise (σ=0.05) to a clean spectrum consisting of 3 Gaussian peaks, overlaid with the top 4 most persistent detected peaks; because only 3 true peaks exist, rank 4 necessarily corresponds to noise. The colors of lines in a, b and c indicate the corresponding rank. (**b**) True peaks (ranks 1–3) exhibit markedly higher persistence than any noise-induced candidate; the rank 4 point (dark circle) lies well below the true peaks, demonstrating easy separability. (**c**) Radar chart projecting the six-dimensional persistence hyper-volumes to 2D. This is useful for tuning the parameter ranges to explore but, because of linear projection, the axis magnitudes do not preserve the true volume ratios seen in panel b. (**d**) A 3D slice of the threshold–width–prominence subspace, with the region in which the rank-1 peak is consistently detected shaded in red.

In this context, spectra are acquired from standardized gamma-ray sources—typically ^137^Cs, ^60^Co, and ^22^Na—and a linear calibration is then applied to convert the known peak positions from channel units to energy units. However, identifying the peaks and determining their centroids is still largely a manual or semi-manual process: even when automated routines are used, close supervision and frequent corrections are often required. Traditional algorithms, including those based on wavelet transforms [6] or built-in functions from mathematical software packages, depend heavily on user-defined hyperparameters such as peak width and inter-peak distance. These methods are highly sensitive to small changes in parameter values, often resulting in inconsistent outcomes: a slight adjustment can lead to missing genuine peaks or, conversely, detecting spurious peaks caused by noise [7,8]. Recent advances have sought to mitigate these issues by either training deep neural networks on raw liquid chromatography–mass spectrometry (LC–MS) [7], or employing statistical modeling to reduce reliance on arbitrary thresholds, validating a Bayesian framework for peak detection on challenging data coming from two-dimensional gas chromatography coupled with time-of-flight mass spectrometry (GC×GC-TOF MS) [2]. Similarly, Kilgour et al. [9] introduced an unsupervised threshold optimization approach tailored to mass spectrometry, significantly enhancing detection reliability across instruments. While these methods mitigate noise and offer improvements in robustness, they either remain highly sensitive to individual hyperparameter settings, or often obscure why a peak is retained or rejected, leaving room for approaches that more transparently quantify feature stability. Motivated by these challenges, our work draws inspiration from the field of topological data analysis (TDA) and the concept of persistent homology, which has been celebrated for its ability to capture the underlying shape of data through so-called barcode plots, that display the interval for which a feature is detected when varying a single filtration parameter; by plotting the ranges in which all features (e.g., peaks in a noisy signal) appear and vanish (the birth–death intervals), barcode plots highlight long-lived, and therefore significant, features [10]. Along this line, Scholkmann and colleagues [1] have presented the Automatic Multiscale Peak Detection (AMPD) algorithm, which considers a peak robust if it remains a local maximum at many scales on periodic data. Although effective in single-parameter scenarios, rigorously extending these ideas to multiple parameters encounters fundamental challenges as barcode plots fully characterize data shape for single-parameter filtrations only while, as shown by Carlsson and Zomorodian [11], no equally simple and complete representation exists for multi-parameter settings. To address this, we introduce a practical framework that aggregates peak detection outcomes across a multi-dimensional hyperparameter space Θ, deliberately simplifying the representation by disregarding detailed shape information. We quantify peak robustness as a scalar measure, defined as the accumulated hyper-volume within the hyperparameter space Θ over which each peak is consistently identified. Concretely, each detected peak corresponds to a specific sub-region of Θ, as shown in Figure 1; panels (b) and (c) in particular show that peaks which appear visually more significant in the raw spectra naturally occupy larger associated hyper-volumes, providing an intuitive basis for ranking stability using their persistence. While exploring an $m^{n}$ hyperparameter space—where *m* represents the number of discretized values per parameter and *n* the number of parameters—can potentially lead to significant computational costs, we effectively mitigate this by employing computationally efficient peak detection routines and extensive parallelization techniques, keeping the runtime of the energy calibration conducted in Section 3.2 at around 40 spectra (each consisting of 2048 channels) per second running the code locally on a laptop. Additionally, our framework is well suited for future enhancements such as adaptive sampling strategies on the parameter space Θ, which can significantly reduce computational overhead by concentrating computational resources in regions of Θ where peak detection outcomes exhibit greater variability.

The remainder of the paper is organized as follows. Section 2 describes our methodology in detail, Section 3.1 presents validation on simulated data, and Section 3.2 demonstrates the application of our method to the real case of the PI3SO spectroscopic data.

## 2. Materials and Methods

The identification of peaks in spectral or time-series data typically requires careful tuning of various hyperparameters. Traditional methods evaluate these parameters individually or in limited combinations, resulting in peak detection outcomes that are highly sensitive to specific settings. Our methodology, inspired by the field of topological data analysis and persistent homology techniques [10,11,12,13], addresses this by introducing the concept of volumetric multi-parameter persistence or VM-PP. Here, the robustness of a detected peak is quantified by systematically exploring and aggregating detection results across a multi-dimensional parameter space.

Formally, we define the hyperparameter space Θ as a discrete grid spanned by the following parameters, each defined as follows:

- Smoothing window size: Number of neighboring data points averaged to smooth short-term fluctuations.
- Bin aggregation factor: Number of adjacent spectral channels combined into one, reducing spectral resolution and noise.
- Prominence: The minimum vertical distance between the peak and the lowest point to which one must descend to reach a higher peak. It quantifies how much a peak stands out from its surrounding landscape—not just its immediate neighbors—in the context of the whole signal.
- Threshold: The minimum vertical difference between the peak and its *immediate neighbors*. Unlike prominence, this is a strictly local criterion: it filters out small fluctuations or noise spikes that do not sufficiently rise above their direct surroundings.
- Width: Required minimum width of detected peaks at half-prominence.
- Peak-to-peak distance: Minimum separation allowed between neighboring peaks.

**Figure 2:**
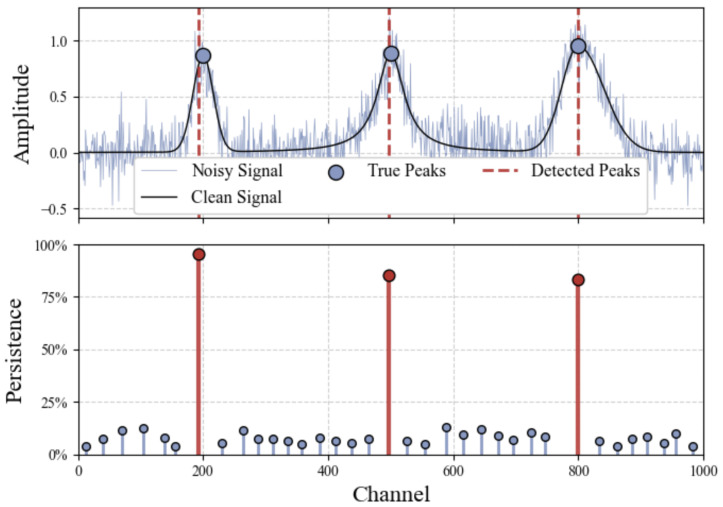
**Top panel**: Simulated test spectra composed of Gaussian, Lorentzian, and asymmetric peaks under intense Gaussian noise (σ=0.15). Vertical lines indicate detected peak channels. **Bottom panel**: VM-PP of peaks expressed as percentage of the total parameter space, allowing peaks that fall below the 25% persistence threshold to be discarded.

**Figure 3:**
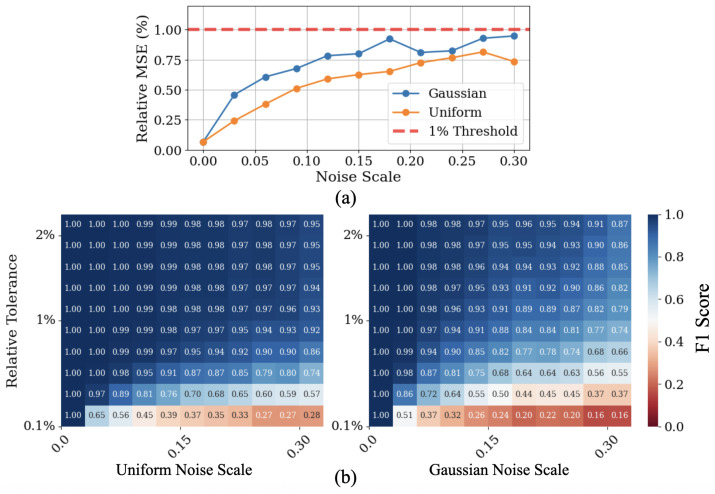
Performance of the multi-parameter persistence approach under varying noise conditions. Panel (**a**) plots the relative mean square error (%) against noise scale for Gaussian (blue) and uniform bounded-range (orange) noise, with a horizontal red line marking the 1% threshold. Panel (**b**) displays maps of detection rate as a function of noise scale (x-axis) and relative tolerance (y-axis) for uniform (left) and Gaussian (right) noise types. Cooler colors in the heatmaps indicate higher detection rates. It is worth underscoring that the ranges explored represent quite heavy noise conditions, as shown in Figure 2, which reports an example spectrum under Gaussian noise with σ = 0.15.

The grid cells in this parameter space correspond to unique parameter combinations, each having equal volume ΔV. While cell volume could be defined proportionally to parameter step sizes, such an approach may disproportionately emphasize parameters explored at a more coarse scale. Thus, for simplicity and fairness, we assign each cell unitary volume, making the persistence of a peak simply equal to the count of parameter cells where it is detected:

(1) $$P\left(f\right)=\sum_{k\in K(f)}\Delta V_{k}$$

where K(f) denotes the set of cells in the parameter space where feature *f* is identified. In other words, under this simplification the method simply measures the fraction of parameter combinations that result in a peak being detected, using this value as a metric proportional to the peak significance. While this may seem a trivial approach, our tests (exemplified visually by Figure 1 and Figure 2) show the remarkable ability of the method to robustly separate genuine peaks from noise-induced fluctuations, minimizing spurious detections when evaluated on simulated signals (Figure 3). Even though in the following we will always apply this simplification, the method remains flexible for future tasks that may benefit from setting specific weights to each explored dimension in order to tune each cell’s volume based on the impact of individual parameters on peak detection. Peak detection within each cell utilizes Python’s scipy.signal.find_peaks function. To enhance computational efficiency, the parameter exploration is structured in two nested grids: an outer grid iterates over preprocessing parameters (smoothing and bin aggregation), while an inner grid extensively covers detection parameters (prominence, width, threshold, and distance). For each outer-grid configuration, data is preprocessed once, and peak detection is executed in parallel across all inner-grid configurations. Detected peaks in closely adjacent positions (within a predefined spatial merging tolerance) are merged and mapped back to their original spectral channels when bin aggregation is applied. Each peak thus acquires a persistence value quantifying its detection robustness across the parameter space. Peaks are ranked according to their persistence scores, which correlate directly with physical significance and detection stability.

To ensure reproducibility, the following parameter ranges were fixed for all applications presented in the paper:

- Smoothing window size: {1,3,5} (1 = no smoothing).
- Bin aggregation factor: {1,2} (1 = no aggregation).
- Prominence: 10 values uniformly spaced from 0.0 to 1.0.
- Threshold: 5 values uniformly spaced from 0.0 to 0.08.
- Width: 7 values uniformly spaced from 1 to 40.
- Distance: 5 values uniformly spaced from 1 to 30.

These choices were the result of a short trial-and-error selection and offer a practical balance between detailed parameter exploration and computational feasibility. Future implementations may employ adaptive selection of the parameter ranges that automatically maximize the difference in persistence between genuine peaks and noise-induced detections, running a preliminary calibration run on a small subset of hand-labeled signals.

For illustrative clarity, Figure 1 panels (c) and (d) visualize this concept explicitly, showing the hyper-volumes occupied by the top-ranked peaks in the parameter space. Specifically, panel (c) shows a linear projection of all 6 explored dimensions that is useful for tuning the ranges of parameters to explore, while panel (d) shows the 3D width–threshold–prominence parameter subspace, with the region occupied by the most persistent peak of the signal in panel (a) colored in red. This approach is able to effectively separate genuine peaks from noise-induced fluctuations even in intense-noise conditions, as shown in Figure 2, where the amplitude of fluctuations has a comparable scale to the clean signal. When the number *k* of expected peaks in a spectrum is known a priori, as is the case for the spectra employed for energy calibration detailed in Section 3.2, one can simply rank all detected peaks by their persistence and take the top *k* most persistent peaks, discarding all others without the need for additional hyperparameters. For more agnostic exploratory tasks where the number of expected peaks is not known, we can select a persistence threshold below which peaks are discarded; the bottom panel of Figure 2 shows that the method effectively separates genuine peaks from noise-induced detections, making the appropriate range for this threshold quite wide, even for very noisy spectra.

To contextualize computational performance, our implementation processes approximately 30 to 50 spectra per second (depending on the resolution and size of the parameter space) using parallel computation on a standard laptop (Python version 3.11.x, tested primarily on macOS Sequoia 15.4 but portable to any platform supporting Python), for spectra consisting of 2048 channels. Despite the exponential scaling inherent to a full $m^{n}$ exploration of the parameter space (with *m* steps per *n* parameters), computational costs can be mitigated; specifically, the use of scipy.signal.find_peaks ensures minimal per-cell runtime, while independent parameter grid cells are evaluated in parallel using multiprocessing or distributed frameworks. Moreover, because the detection function is piecewise continuous (Appendix A.1) over the parameter space, adaptive sampling strategies can be employed in future implementations to reduce the number of necessary evaluations. The methodology described has been fully implemented in Python, leveraging parallelization libraries to ensure efficiency. To foster reproducibility and support the scientific community, our implementation, along with an interactive demonstration, is publicly accessible via GitHub [14].

## 3. Results

The following section is divided into two parts: in the first, we benchmark our method on synthetic data and evaluate its performance when varying noise conditions; in the second, we showcase an application of our method to real spectroscopy data.

### 3.1. Synthetic Data Benchmark

To evaluate the robustness of our multi-parameter persistence method under controlled conditions, we generated a synthetic dataset consisting of 5000 test spectra. Each test spectrum contained three distinct peaks: one Gaussian, one Lorentzian, and one asymmetric, carefully positioned to avoid overlap. The amplitudes of these peaks were normalized so that each clean (noise-free) spectrum remained within the range (0,1), ensuring that no individual peak exceeded an amplitude of 1.

To simulate realistic measurement conditions, we introduced two types of noise separately into each test spectrum:

- Uniform noise, where each data point was randomly perturbed by an amount between −a and +a, with the noise amplitude *a* varied from 0 to 0.3.
- Gaussian noise (additive white Gaussian noise; AWGN), where each data point was perturbed by a value drawn from a Gaussian distribution with mean 0 and standard deviation σ varied from 0 to 0.3.

An example synthetic spectrum with moderate Gaussian noise (σ=0.15) is shown in Figure 2. Detected peaks are marked by vertical dashed lines. The bottom panel illustrates the peak persistence scores, clearly showing how the three true peaks stand out significantly from random noise fluctuations.

The performance of our detection method across varying noise levels is summarized in Figure 3. To quantify detection accuracy clearly, we defined two separate metrics:

- Relative mean detection error: For each detected peak, we measured the absolute difference between the predicted channel (detected peak position) and the actual known peak position. This error was normalized relative to the full range of the spectrum to produce a percentage error. We then averaged this error over all three peaks in each spectrum and over all 5000 test spectra for each noise level. Panel (a) displays this averaged error metric, showing how detection accuracy degrades with increasing noise.
- Detection rate (F1 score): To assess how reliably peaks were found, we used a common classification metric known as the F1 score. This metric combines two important aspects into a single value:
  1. Recall: The fraction of actual peaks successfully detected.
  2. Precision: The fraction of detected peaks that corresponded to actual (rather than noise-induced) peaks.
  The F1 score is defined as the harmonic mean of recall and precision and reaches its maximum value of 1.0 (100%) when all true peaks are detected correctly without false positives. For instance, an F1 score of 0.98 indicates that overall about 2% of detections were incorrect—either missed true peaks or included spurious noise peaks.
  Panel (b) of Figure 3 shows the F1 score for varying noise levels and different tolerance values. The relative tolerance refers to how close a detected peak must be to the true peak position to be considered a correct detection, again expressed as a percentage of the full spectral range. Darker blue areas in the heatmaps indicate excellent detection performance (close to 1), whereas lighter (warmer) colors indicate lower reliability.

The heatmaps demonstrate that our approach maintains strong performance under substantial noise. The uniform noise scenario (panel b) consistently showed excellent performance, while the Gaussian noise scenario (panel c) exhibited slightly lower but still robust detection rates, reflecting the more challenging nature of unbounded Gaussian fluctuations.

In summary, the synthetic tests clearly demonstrate that our multi-parameter persistence approach reliably detects and accurately locates peaks even under high-noise conditions. The results underscore both the method’s ability to consistently identify all peaks (high recall) and avoid false detections (high precision), qualities that directly translate into practical advantages when applied to experimental spectroscopic data.

### 3.2. Validation on Spectroscopy Data

To validate the effectiveness of our peak detection and classification methodology, we utilized experimental gamma-ray spectroscopy data obtained using the Proximity Imaging System for Sort and Segregate Operations (PI3SO) [3]. This advanced spectroscopic system automates the identification of gamma-ray-emitting sources within radioactive waste, specifically targeting intermediate-level waste (ILW), low-level waste (LLW), and very low level waste (VLLW) [4,5]. The PI3SO system can improve radwaste (re)conditioning and management by rapidly identifying radioactive hotspots with minimum human intervention.

#### 3.2.1. PI3SO Spectroscopic System

**Figure 4:**
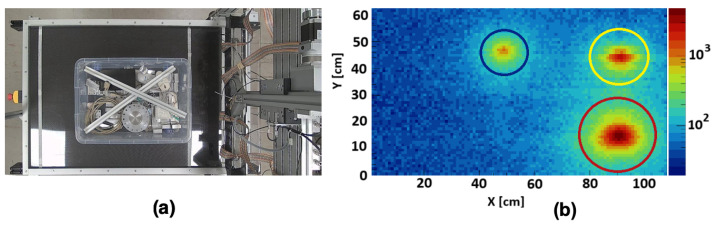
(**a**) System operation diagram: Radioactive waste is distributed over the scanning table to identify radioactive hotspots. (**b**) Visualization of the result of a scan of three different radioactive point-like sources; color scale on the right reports the activity in cps. Red circle: source of ^137^Cs, with an activity of 1.4000 MBq; yellow circle: source of ^60^Co, with an activity of 0.0560 MBq; blue circle: source of ^22^Na, with an activity of 0.0154 MBq.

The PI3SO project primarily addresses two interconnected tasks: Hotspot search and gamma spectrometry. Hotspot search involves identifying areas containing radioactive objects (see Figure 4a). Once identified, these hotspots become regions of interest (ROIs) for subsequent detailed gamma spectroscopic analyses, as illustrated in Figure 4b.

**Figure 5:**
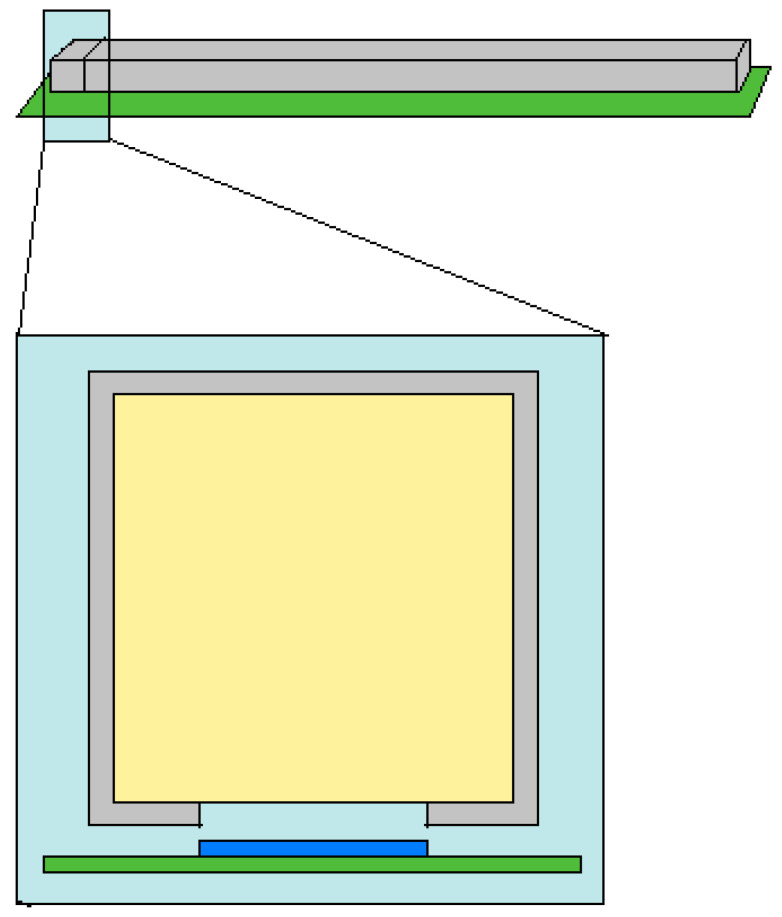
**Top**: Sketch of a single component of one of the two linear arrays, which consists of four modules like the one shown, placed next to each other in a row; each module houses a total of 16 CsI(Tl) crystals embedded in a reflective resin case and coupled to as many SiPMs that are housed on an electronic board. **Bottom**: Sketch of a single detector: In gray is the reflective mask in which the 1 × 1 × 1 cm^3^ crystal of CsI(Tl) (in yellow) is embedded; the latter is optically coupled through its free face to a 6 × 6 mm^2^ SiPM (blue), using optical grease with intermediate refractive index to minimize reflection losses at the interface. The SiPM is housed in the electronic board (green).

The PI3SO instrumentation consists of a robust table coupled with a mechanical sliding bridge. Two linear arrays of 64 gamma-ray detectors, are arranged above and beneath the table. Each array contains four modules of 16 detectors, each detector coupling a cubic (1 cm^3^) CsI(Tl) scintillator with a silicon photomultiplier (SiPM). The detectors are encapsulated in reflective casings, enhancing photon collection efficiency. The structural layout and module configuration are detailed in Figure 5.

CsI(Tl) crystals were chosen for their optimal properties, including high density (4.51 g/cm^3^), superior energy resolution, and significant light yield (60,000 photons/MeV), with a peak emission around 550 nm [15,16]. Each scintillator is coupled to an MPPC (multi-pixel photon counter) SiPM from Hamamatsu Photonics K.K. (325-6, Sunayama-cho, Chuo-ku, Hamamatsu City, Shizuoka Pref., 430-8587, Japan), featuring an active area of 6 × 6 mm^2^ and 14,331 cells [17]. Previous tests confirmed an energy resolution (FWHM) around 5–6% for the entire detection system [3]. Signals from each detector are digitized using two VX2745 digitizers [18], produced by CAEN SpA (Via Vetraia 11, 55049 Viareggio, Italy) each one capable of simultaneously sampling signals from 64 SiPM channels at 125 MSamples/s with a 16-bit resolution. Data acquisition is synchronized via timestamps, allowing precise temporal alignment of detected events. The acquisition system, interfaced via Ethernet, supports real-time data analysis, spectral generation, and comprehensive event logging. During typical calibration or operational measurements, nearly all 128 detectors are actively engaged, generating extensive datasets. Each dataset typically comprises multiple gamma-ray spectra requiring energy calibration and isotope identification. Given the complexity and volume of the data, manual analysis is impractical. Consequently, an automated, robust peak identification system is essential.

Our validation procedure leveraged detector calibration tasks involving three standardized radioactive sources: ^137^Cs (1.4 MBq, 0.662 MeV), ^60^Co (0.056 MBq, 1.173 MeV, and 1.331 MeV), and ^22^Na (0.015 MBq, 0.511 MeV, and 1.274 MeV). Each of the 128 detectors was exposed to these sources individually to validate automated peak detection and classification without manual intervention.

#### 3.2.2. Peak Ranking and Automatic Selection

**Figure 6:**
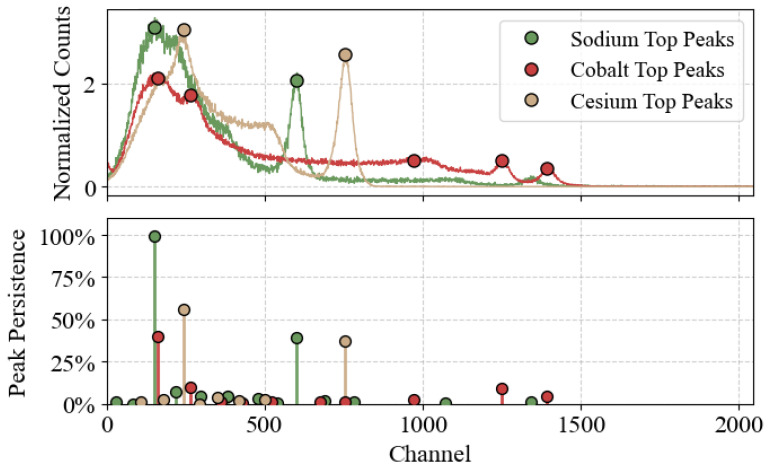
VM-PP applied to peak detection on 3 sample sources: **Top panel** shows the emission spectra of sodium, cobalt, and cesium sources, normalized by area under curve and annotated with the top *k* most persistent peaks for each source; **bottom panel** shows the persistence of detected peaks, reported as the percentage of volume in parameter space where the peak is detected. For all 3 spectra, despite significant differences in peak shapes, the same portion of parameter space is explored, showcasing the versatility and robustness of our method.

Figure 6 illustrates the application of our VM-PP method to typical emission spectra from sodium, cesium, and cobalt radioactive sources, for which the noise scale was estimated to be between σ=0.05 and σ=0.10 (lower noise scales could be achieved at the cost of increasing the exposure time), highlighting the robustness of the peak-ranking process across spectra with varying complexities. Using our multi-parameter persistence ranking, we select from each detector the top *k* most persistent peaks. The choice of *k* depends on the source’s spectral shape, but requires almost no additional filtering.

- For sodium, we set k=2 to catch both the main emission peak at 0.511 MeV and a secondary peak at lower energy, attributed to backscattered photons. This peak originates from gamma rays that escape the detector without interacting and are scattered backward (at angles close to 180°) by surrounding materials, and then re-enter the detector with reduced energy (note that this is not the Compton edge, which corresponds to the maximum energy transferred to an electron during a single Compton scattering event).
- For cesium, k=2 again recovers the main peak (0.662 MeV) plus a secondary region at lower energy.
- For cobalt, k=5 covers the overall complexity of the spectrum well; the two highest energy peaks of those five correspond to the known 1.17 and 1.33 MeV lines.

**Figure 7:**
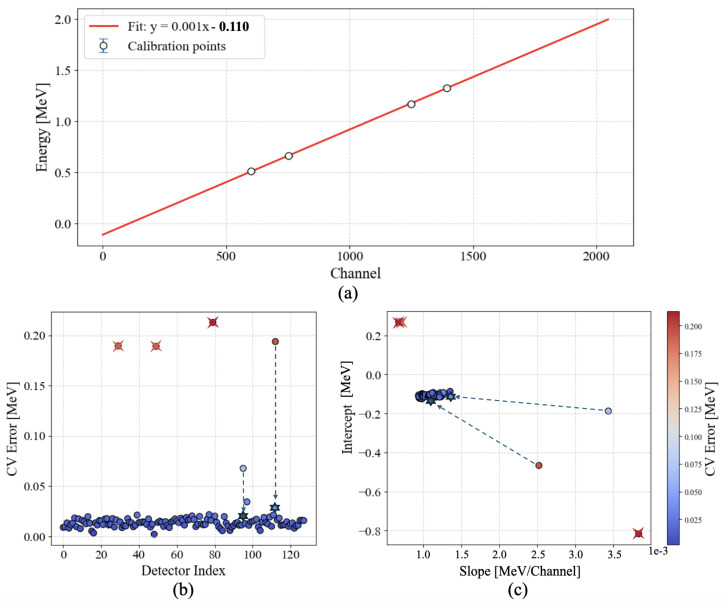
Calibration results: (**a**) An example linear best fit on a single detector’s data, using 4 emission peaks of known energy from sodium (0.511 MeV), cesium (0.662 MeV), and cobalt (1.17 and 1.33 MeV). (**b**) The leave-one-out (LOO) cross-validation (CV) error of each detector’s calibration, highlighting 3 outlier points resulting from faulty detectors (crossed circles). The 2 remaining points with poor performance (circles) are easily recovered with minimal post-processing, the results of which are shown as stars. (**c**) Distribution of best-fit slope and intercept parameters across all detectors, colored by the LOO-CV error. Negative intercept values arise from well-known non-linearities near the extremes of the detectors’ ranges.

Initially, three detectors were excluded from the analysis due to severe hardware damage, clearly identified by significant deviations in their calibration fits (Figure 7b,c). Among the remaining 125 detectors, the correct calibration peaks were identified directly within the top *k* sets for 123 cases without any additional processing. However, in two detectors, the peaks of interest were ranked just outside the top *k* sets, appearing as the (k+1)-th most persistent peaks. We found that minimal additional filtering—such as applying a broad energy/channel cut to eliminate clear backscattering peaks—was sufficient to recover these peaks accurately. Ultimately, using the persistence-based ranking approach combined with this minor post-processing step in the two challenging instances, we successfully identified all four targeted emission peaks across the full set of 125 operational detectors.

#### 3.2.3. Calibration Outcomes

Once the correct peak positions are identified in channel space, a simple linear fit converts channel number to energy. The fit uses the known energies of each peak as the *x* values and the measured channel positions as the *y* values. In Figure 7, we illustrate the overall calibration performance.

Panel (a) of Figure 7 shows a typical example of a linear energy calibration fit for a single detector, using the four known gamma-ray emission peaks. To evaluate the reliability of these calibrations across all detectors, we employed a standard technique known as leave-one-out cross-validation (LOO-CV). In this procedure, the calibration is repeated four times per detector, each time leaving out one of the four reference peaks, fitting a line to the remaining three, and then computing the prediction error on the omitted point. Panels (b) and (c) summarize the results of this analysis across the full detector array. For each detector, the individual prediction errors from the four LOO-CV iterations are averaged to yield a single scalar error value. These average errors are reported in panel (b) as a measure of calibration reliability and are also used to color-code the corresponding slope and intercept values in panel (c). Lower average error indicates that all four calibration peaks lie close to a consistent linear fit, while higher error suggests issues such as misidentified peaks or detector faults. In particular, the three marked detectors with extremely high errors were identified as irreparably damaged and removed from subsequent analyses. The remaining two elevated-error points arise from the two detectors in which the primary cobalt peaks initially ranked as (k+1)-th. Because the correct peaks are nonetheless detected (just slightly lower in the persistence order), a mild filter on backscattering peaks fixes the issue.

Panel (c) in Figure 7 summarizes how the fit parameters vary across the detectors. Most calibrations cluster around consistent slope/intercept values, with small variations attributed to manufacturing differences, gain settings, or mild hardware non-linearities. Negative intercepts persist in all detectors, reflecting known edge effects and electronic offsets but still yielding an accurate mapping over the region of interest.

#### 3.2.4. Channel-to-Energy Translations

**Figure 8:**
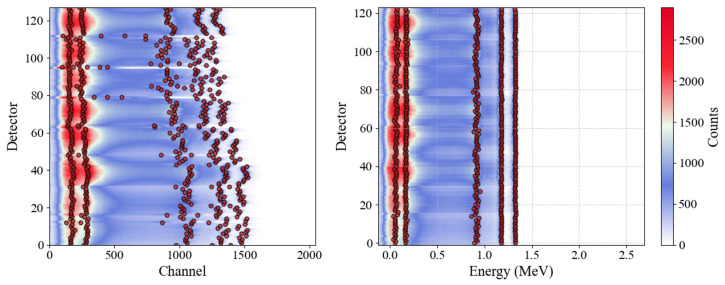
Example cobalt emission spectra from all detectors, annotated with the top 5 most persistent peaks. (**Left**): raw data in channel space reveal large variability in apparent peak locations. (**Right**): the same spectra after linear calibration and removal of 3 faulty detectors, translating channel to energy. The highest two (energy-wise) of these 5 persistent peaks correspond precisely to the known 1.17 MeV and 1.33 MeV emissions. The triangular-shaped shadows visible in the region around 0.3 MeV with a periodicity of 16 detectors depend on the different counting rates due to different center-to-side solid angles when placing the point-like source in front of each 16-unit detector module.

To visualize the overall outcome of this multi-detector calibration, we compare raw (channel-space) spectra to their energy-space equivalents. Figure 8 shows, for a cobalt source, how the large spread of peak positions among the 128 detectors (left) collapses into well-aligned peaks once the linear transformations are applied and the 3 faulty detectors removed (right).

By ranking peaks via multi-parameter persistence, each detector automatically yields the relevant peaks. Even small deviations in detection (as for the two problematic detectors mentioned above) are trivially corrected by discarding the low-energy backscattering regions. Hence, without ever manually labeling any peak, we successfully identify all calibration peaks for 125 detectors, requiring minimal filtering to recover even the two initially mislabeled peaks. This amounts to detecting (and correctly matching) 4×125 = 500 emission peaks across all spectra, confirming the robustness of the proposed persistence-based approach for automating detector calibration.

## 4. Discussion

The proposed multi-parameter persistence framework demonstrates substantial advantages in robustness, interpretability, and practical utility for peak detection across both simulated and real-world datasets. The methodology generalizes traditional single-parameter persistence concepts, quantifying the stability of detected features by integrating across multiple influential detection parameters such as prominence, distance, width, and threshold. This comprehensive approach significantly mitigates the sensitivity to arbitrary parameter selection, an inherent limitation of conventional peak detection algorithms. Validation on test datasets (Section 3.1) underscores the robustness of this approach. Our experiments revealed consistently low detection errors—under 1%—across a wide range of noise intensities, including challenging scenarios with both Gaussian and uniform noise distributions. Heatmap analyses further confirmed the method’s resilience, highlighting expansive parameter regions where near-perfect detection rates were consistently achieved, thus establishing its reliability under various practical conditions. When applied to real-world spectroscopic data for detector calibration (Section 3.2), the method’s effectiveness was clearly demonstrated. Using automated, persistence-based peak ranking without manual labeling, all critical calibration peaks across a large detector array were successfully identified. The framework proved not only capable of accurately calibrating functional detectors but also adept at diagnosing and isolating hardware faults, streamlining the instrument maintenance process. In cases where the automated method initially ranked correct peaks slightly lower in persistence, minimal post-processing sufficed for full recovery, highlighting the practicality and flexibility of the approach.

An important strength of this method lies in its intuitive interpretability. By assigning each detected feature a clear scalar metric—the accumulated hyper-volume across the parameter space—practitioners gain a straightforward and quantitative measure of peak robustness. This metric inherently simplifies the feature-ranking process, distinguishing meaningful peaks from noise-induced artifacts effectively and transparently. From a theoretical standpoint, this work bridges traditional signal-processing techniques with concepts from multi-parameter persistent homology (MPH). While formal MPH can offer meaningful insights by analyzing data through intricate topological invariants, our simpler volumetric persistence measure provides an accessible yet powerful alternative that is both computationally efficient and easy to interpret. Future research could further explore adaptive or probabilistic sampling strategies to optimize computational efficiency or integrate formal MPH invariants to uncover additional structural insights in complex multi-dimensional datasets.

In conclusion, the multi-parameter persistence method introduced here represents a robust, versatile, and intuitive solution to feature detection tasks across a variety of applications. Its successful application in simulated and real-world spectroscopic scenarios demonstrates its broad potential, offering enhanced reliability, reduced parameter sensitivity, and clear interpretability that are essential for modern scientific and engineering analyses.

## Data Availability

The spectroscopy data analyzed in this study were provided under license by the INFN-Laboratori Nazionali del Sud and are not publicly available. The complete Python implementation of the multi-parameter persistence peak detection method introduced in this study is openly available at https://github.com/gullo97/Volumetric-MPP (accessed on 23 July 2025). An interactive online demonstration of the method, allowing users to test it on custom synthetic data, as well as user-provided signals, will also be publicly accessible at the same repository upon publication.

## Abbreviations

The following abbreviations are used in this manuscript:

LOOCV	Leave-one-out cross-validation
MPP	Multi-parameter persistence
LC–MS	Liquid chromatography–mass spectrometry
GC×GC-TOF MS	2D gas chromatography with time-of-flight mass spectrometry
VM-PP	Volumetric multi-parameter persistence

## Appendix A

### Appendix A.1. Piecewise Continuity of the Detection Function

In this section we establish that, under reasonable assumptions, the detection function used in our methodology is piecewise continuous. Let $\Theta \subset R^{n}$ denote the hyperparameter space and consider the function

f:Θ→{0,1},

where f(θ)=1 indicates that a particular feature (e.g., a peak) is detected for the hyperparameter setting θ, and f(θ)=0 otherwise.

We assume that the underlying data function D(x) is continuous and that the detection of a feature at a candidate location $x_{0}$ is determined by inequalities of the form

$$D\left(x_{0}\right)\geq \tau \left(\theta \right)\;\mathrm{and}\;D\left(x_{0}\right)-D\left(x\right)\geq \delta \left(\theta \right)$$

for *x* in a neighborhood of $x_{0}$, where τ:Θ→R and δ:Θ→R are continuous functions representing, respectively, a detection threshold and a margin requirement.

Suppose first that for some $\theta_{0}\in \Theta$ the detection conditions are satisfied (i.e., $f(\theta_{0})=1$). By the continuity of τ and δ, there exists an open neighborhood U⊂Θ containing $\theta_{0}$ such that the inequalities

$$D\left(x_{0}\right)\geq \tau \left(\theta \right)\;\mathrm{and}\;D\left(x_{0}\right)-D\left(x\right)\geq \delta \left(\theta \right)$$

continue to hold for all θ∈U. Hence, f(θ)=1 for all θ∈U. Conversely, if $f(\theta_{0})=0$, then the failure of the detection condition persists in an open neighborhood V⊂Θ about $\theta_{0}$, so that f(θ)=0 for all θ∈V.

Thus, aside from the boundaries where the detection outcome changes, the function *f* is constant—and therefore continuous—on open subsets of Θ. The discontinuities, if any, are confined to the boundaries between these open regions, which are typically of lower dimension. In this sense, the detection function *f* is piecewise continuous.

This property is essential for justifying adaptive sampling strategies. In regions where *f* is continuous, a coarse sampling suffices to capture its behavior, while finer resolution can be concentrated near the transition boundaries, thereby mitigating the computational burden of a uniform grid search.

### Appendix A.2. Connection to Multi-Parameter Persistent Homology

From a TDA perspective, detecting and tracking features across an *n*-dimensional parameter space is akin to multi-parameter persistence. Classical persistence homology often focuses on a single parameter (e.g., a filtration value), but Θ here involves multiple dimensions—leading to more complex invariants in multi-parameter persistent homology (MPH) [13]. In principle, one could model feature detections themselves (e.g., peaks) as 0D topological features that appear or vanish across the hyperparameter lattice.

Our extension of “persistence” to a higher-dimensional hyperparameter domain is conceptually aligned with MPH, yet we opt for a simpler, domain-specific aggregator: the accumulated hyper-volume in which a feature is detected. This single scalar score captures the same spirit of “robustness under parameter variation” without the overhead of constructing a full multi-parameter barcode. Its advantages are as follows:

- Straightforward to compute: A grid search (or analogous sampling) across the parameter space directly yields the measure of persistence for each feature.
- Intuitive to interpret: It quantifies how “hard” it is to destroy a feature by shifting the algorithm’s hyperparameters.
- Avoids complexity: Formal MPH approaches may require the identification of complex topological invariants, which can lead to intricate representations of the data and high computational overhead.

In the TDA literature, advanced tools like bigraded Betti numbers or rank functions can become unwieldy in higher dimensions, because multi-parameter persistence is known to be “wild” from a representation-theoretic perspective [10,11,12,13]. By contrast, our aggregated volume measure is easy to compute and well suited to routine signal-processing tasks. Nevertheless, an interesting future direction is to compare our hyper-volume aggregator with more formal MPH tools to see whether further insights or additional topological structures might be revealed. We view this approach as a simple, application-driven extension of persistence ideas into robust feature detection while still connecting to the broader multi-parameter TDA framework [11].
